# Architecting Phase‐Selective Interlayers for Stable SiO_x_ Lithium Storage via Dual‐Function Design

**DOI:** 10.1002/advs.77220

**Published:** 2026-08-19

**Authors:** Zhenfei Chang, Wei Dong, Yingguang Zhang, Hangchen Qu, Yifei Wang, Xiaolong Zhao, Chu Wang, Ming Dong, Huizhi Wang, Jin Xuan, Yug Joshi, Dennis Y.C. Leung, Wending Pan

**Affiliations:** ^1^ School of Energy and Power Engineering Dalian University of Technology Dalian Liaoning Province China; ^2^ Department of Mechanical Engineering The University of Hong Kong Hong Kong Hong Kong; ^3^ Department of Mechanical Engineering Imperial College London London UK; ^4^ School of Chemistry and Chemical Engineering University of Surrey Guildford UK; ^5^ Max‐Planck Institute for Sustainable Materials Düsseldorf Germany

**Keywords:** bulk deoxygenation, conductive wiring, lithium silicate phase selection, phase‐selective conductive interfacial interlayer, SiO_x_ anodes

## Abstract

Silicon suboxide (SiO_x_) anode offer higher capacities than graphite; however, their practical use is hindered by unstable interfaces and poorly controlled formation of lithium silicates during initial lithiation. Insufficient or nonuniform lithiation results in oxygen‐rich lithium silicates that block deep silicon (Si) alloying, while uncontrolled interfacial reaction consumes active lithium, lowers initial Coulombic efficiency, and destabilizes the solid‐electrolyte interphase (SEI). In this study, we address a specific, falsifiable question: what factors dictate which lithium silicate phase forms in the subsurface, and can this process be manipulated to improve both deep Si alloying and SEI stability? We define the Phase‐Selective Conductive Interfacial Interlayer (PSCII) as a Li_4_SiO_4_‐rich and ion‐accessible subsurface formed through coupled control of bulk oxygen stoichiometry and local electron supply. A stepwise control series from commercial SiO to layered SiO_x_ (LS‐SiO_x_), deoxygenated layered SiO_x_ (DLS‐SiO_x_), and single‐walled carbon nanotubes (SWCNTs)‐wired DLS‐SiO_x_@CNT separates the contributions of layered buffering, bulk deoxygenation, and conductive wiring. As a result, DLS‐SiO_x_@CNT delivers 1650 mAh g^−1^, retains >820 mAh g^−1^ after 700 cycles at 1 A g^−1^, and enables stable LCO full‐cell cycling. These results identify subsurface lithium silicate phase selection as a practical design strategy for durable high‐capacity SiO_x_ anodes.

## Graphical abstract

Silicon anodes offer high capacity but suffer severe volume expansion. Silicon monoxide (SiO) buffers expansion, yet uncontrolled lithiation irreversibly consumes lithium as Li_2_O and oxygen‐rich silicates. Here, we design a layered SiO_x_ featuring a phase‐selective conductive interfacial interlayer (PSCII), i.e., a Li_4_SiO_4_‐rich, ion‐accessible subsurface that controls lithium consumption, enables deep Si alloying, and stabilizes the SEI for durable, high‐capacity cycling.

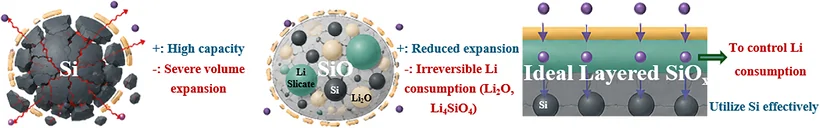

## 1 Introduction

The pursuit of higher‐energy lithium‐ion batteries begins with the limitations of graphite, whose theoretical capacity is only 372 mAh g^−1^ [1, 2, 3]. Elemental Si offers an order‐of‐magnitude higher capacity because it can alloy with Li to form Li‐rich phases approaching Li_15_Si_4_, but this advantage is coupled to an extreme reaction extent: deep lithiation causes large volume expansion, particle fracture, continuous SEI rupture/reformation, and severe first‐cycle lithium loss [4]. The central challenge for Si‐based anodes is therefore not simply to make Si react more, but to control how far, where, and how uniformly the first lithiation reaction proceeds. If the reaction is spatially uncontrolled, high capacity is obtained at the expense of interfacial stability and initial Coulombic efficiency (ICE).

SiO_x_ emerged as a practical compromise between graphite and elemental Si [5]. By incorporating oxygen into the Si framework, SiO_x_ reduces the effective volume change and generates Li_2_O/Li_x_Si_y_O_z_ species during the first lithiation that can act as an internal buffer [6, 7]. This built‐in buffering explains why SiO_x_ is often more cyclable than pure Si, but it also creates the defining penalty of SiO_x_: irreversible lithium consumption and the formation of electrochemically inactive or transport‐blocking lithium silicates lower both ICE and accessible capacity. In other words, the benefits and drawbacks of SiO_x_ arise from the same first‐cycle chemistry [8, 9]. Zhang et al. demonstrated this principle by constructing a core‐shell Si/SiO nanocomposite via the sol‐gel method, where the SiO shell served as a physical barrier to prevent Si nanoparticle aggregation during cycling while simultaneously forming lithium silicates that buffered volume expansion [10]. The key variable is the extent of first‐lithiation reaction of SiO_x_: insufficient or nonuniform reaction leaves oxygen‐rich, Li_2_Si_2_O_5_‐like blocking chemistry that suppresses deep Si alloying, whereas excessive exposure of reactive interfaces drives electrolyte decomposition and low ICE [11]. A useful SiO_x_ anode must therefore regulate the extent and spatial uniformity of first lithiation so that active Si can be utilized deeply while parasitic interfacial reactions are limited.

Existing modification strategies address parts of this problem but rarely control this phase selection directly. Carbon coatings [12, 13, 14], graphene wrapping [15, 16, 17], and nanotube additives [17, 18, 19] improve electronic conductivity yet often act mainly as external conductive shells and do not determine which lithium silicate forms inside the cycled subsurface [20]. Nanostructuring SiO_x_ into spheres [21, 22], wires [23, 24], or porous networks [25, 26] can reduce mechanical fracture, but the larger exposed surface area increases electrolyte side reactions, lowers the initial ICE [27], and can compromise volumetric energy density [28, 29]. Layered SiO_x_ microparticles represent an important advance because disconnected interstices can buffer volume change and restrict electrolyte penetration [30, 31, 32, 33, 34, 35]. Nevertheless, layered architecture alone does not tune the bulk oxygen stoichiometry or guarantee uniform local electron supply during first lithiation [36, 37, 38]. As a result, the subsurface chemistry, deep Si alloying accessibility, and long‐term SEI stability remain coupled but insufficiently controlled [32, 36, 39, 40]. In this context, non‐equilibrium techniques such as intense pulsed light (IPL) and high‐temperature shock (HTS) have enabled new routes for electrode structural engineering. Reslan et al. showed that IPL converts Si nanoparticles into graphene‑embedded SiC heterostructures with improved cycling stability and rate capability, while Tao et al. reviewed HTS for defect, phase, and interface engineering via ultrafast Joule heating. These physical‑thermal approaches, however, require specialized equipment and operate under extreme conditions; we instead explore a chemically driven structural design that is more accessible and scalable [41, 42].

**FIGURE 1:**
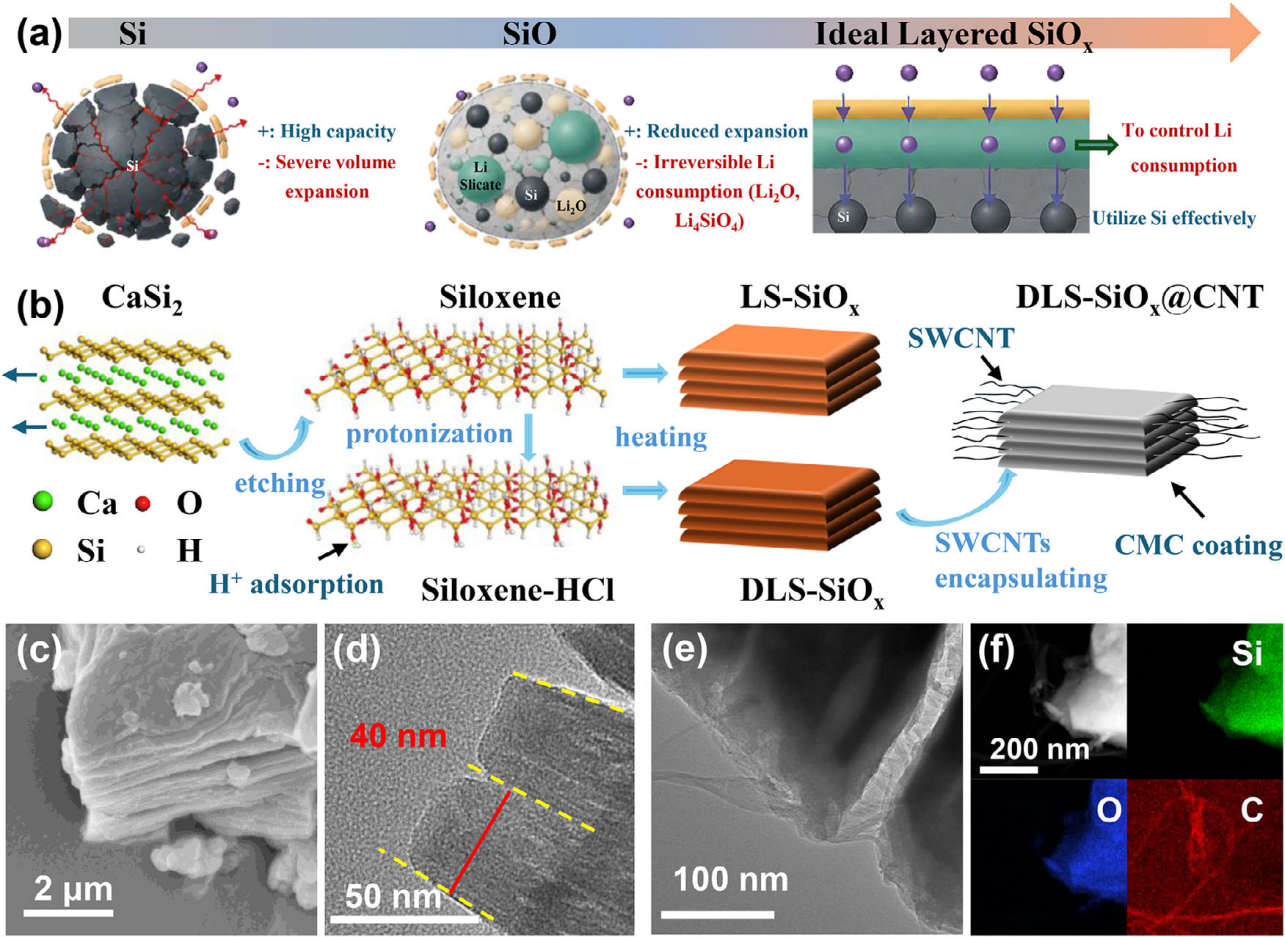
Concept and design framework of coupled control of oxygen stoichiometry and electron supply. (a) Schematic framing of Si, conventional SiO, and ideal layered SiO_x_, emphasizing that the key design problem is to control subsurface lithium silicate formation while retaining access to active Si. (b) Synthesis flow for HCl‐mediated deoxygenation and SWCNT/CMC wiring. (c) SEM image and (d) TEM image of DLS‐SiO_x_. (e) TEM image of DLS‐SiO_x_@CNT. (f) EDS mapping of DLS‐SiO_x_@CNT.

Here, we frame the work around a coupled design that activates the relevant features stepwise (Figure 1a): commercial SiO contains neither design variables; LS‐SiO_x_ introduces layered mechanical buffering; DLS‐SiO_x_ adds HCl‐mediated bulk deoxygenation as a thermodynamic variable; and DLS‐SiO_x_@CNT adds a percolating SWCNT network to regulate local electron supply at the interface. We propose the Phase‐Selective Conductive Interfacial Interlayer (PSCII) as a working hypothesis: the Li_4_SiO_4_‐rich, transport‐permissive, electronically connected subsurface that forms when the first‐lithiation reaction‐extent regulation is jointly achieved by bulk oxygen stoichiometry and interfacial electron supply. This framework predicts that improved interface regulation should raise ICE, suppress Li_2_Si_2_O_5_‐rich blocking chemistry, enable deeper access to the Li_4_SiO_4_‐associated alloying pathway, and stabilize SEI growth. To test these linked predictions, we employed the ICE/capacity evolution, galvanostatic intermittent titration technique (GITT) signatures near 0.35‐0.40 V, XPS depth profiling of lithium silicate phases, and cross‐sectional TEM/EDX of the cycled interface. The experimental results consistently support the proposed hypothesis, with the DLS‐SiO_x_@CNT anode demonstrating exceptional and balanced electrochemical properties, including high specific capacity (>1600 mAh g^−1^), outstanding rate capability (retention >50% at 5A g^−1^), and unprecedented cycling stability (>600 mAh g^−1^ after 700 cycles).

## 2 Results and Discussion

### 2.1 Design and Pre‐Structuring of the PSCII Precursor

Realizing the PSCII is approached through two coupled design variables rather than a generic composite strategy (Figure 1b). The first variable is bulk thermodynamics: HCl‐mediated oxygen reduction lowers the SiO_x_ oxygen stoichiometry and biases first‐lithiation products away from Li_2_Si_2_O_5_‐rich blocking chemistry. The second variable is interfacial kinetics: a conformal SWCNT network supplies electrons uniformly across the layered particles, reducing localized polarization during lithiation. The sample series functions as a control series in which layered buffering, deoxygenation, and conductive network formation are activated sequentially.

The synthesis begins with commercial calcium disilicide (CaSi_2_, Figures S1 and S2). Controlled HCl etching converts CaSi_2_ into layered siloxene while preserving the micrometer‐scale layered morphology (Figure 1b and Figure S3a,c). The additional HCl treatment is used not only to retain the layered scaffold but also to initiate the oxygen‐stoichiometry tuning required for later phase selection. TEM images confirm abundant interlayer voids within a coherent framework (Figure S3b,d), providing the structural buffer needed to accommodate cycling strain.

Thermal annealing of the modulated siloxene precursor yields DLS‐SiO_x_. For comparison, LS‐SiO_x_ is synthesized by direct calcination without the HCl‐mediated deoxygenation step. Both materials inherit microscale layered architecture (Figure 1c and Figure S4a), and high‐resolution TEM shows individual SiO_x_ layers with a confined thickness of approximately 30–40 nm (Figure 1d and Figure S4b). The layered and intralayer porosity shortens diffusion lengths and provides free volume for lithiation‐induced strain.

To activate the electronic wiring variable, SWCNTs were conformally anchored on DLS‐SiO_x_ with sodium carboxymethyl cellulose (CMC), forming DLS‐SiO_x_@CNT. Microscopy shows that nanotube incorporation preserves the layered morphology while creating a percolating 3D conductive network across particle surfaces and interlayer gaps (Figure 1e and Figure S5a). The observed nanotube bundles (Figure S5b) support continuous electronic pathways rather than serving as the primary Li^+^ transport channels. EDS mapping confirms homogeneous Si, O, and C distributions in the composite (Figure 1f). This pre‐assembled SWCNT network couples local electron supply to Li^+^ flux at the developing PSCII and limits localized polarization that would otherwise favor incomplete lithiation and insulating silicate accumulation.

**FIGURE 2:**
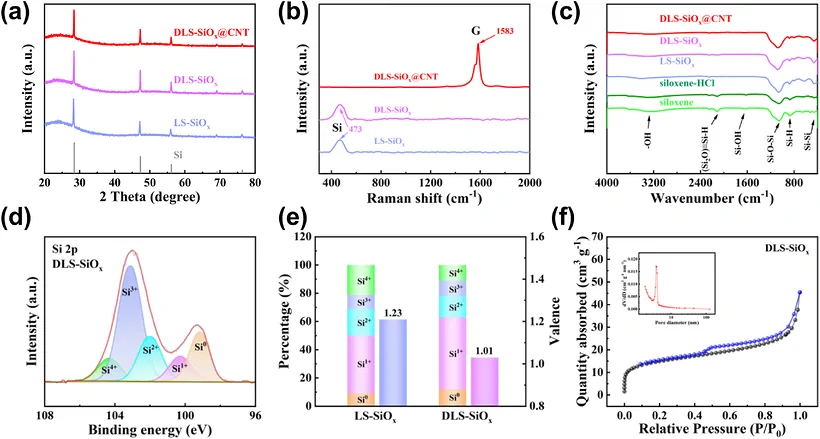
Material‐level verification of the control variables. (a) XRD patterns confirming amorphous SiO_x_ formation and preserved c‐Si. (b) Raman spectra showing the redshifted Si‐related band and the D/G bands after SWCNT incorporation. (c) FTIR spectra showing the HCl‐induced Si–OH protonation step. (d) Si 2p XPS deconvolution (after Ar^+^ sputtering to a depth of 100 nm) and (e) oxidation‐state/Si/O quantification, showing the decrease in oxygen stoichiometry from x = 1.23 to x = 1.01. (f) N_2_ sorption/pore‐size analysis with electronic conductivity comparison.

The dual control variables have been physically engaged in Figure 2. The layered SiO_x_ framework and c‐Si domains are preserved, the oxygen stoichiometry is lowered, and the electronic network is introduced without collapsing the porosity. XRD confirms the conversion from layered precursors to the target amorphous SiO_x_ framework while preserving crystalline Si domains inherited from the CaSi_2_ precursor (Figure 2a and Figure S6). The characteristic siloxene peaks disappear after annealing, whereas LS‐SiO_x_ and DLS‐SiO_x_ exhibit broad halos in the 20–30 degrees range, consistent with amorphous SiO_x_ [43]. Raman spectra further show a broad Si‐related band near 480 cm^−1^ for DLS‐SiO_x_ and the D/G bands of carbon after SWCNT encapsulation (Figure 2b, the Raman of their layered precursors can be found in Figure S7), confirming that an electron percolation network is added while the SiO_x_ framework remains structurally intact.

FTIR spectroscopy identifies the chemical step responsible for oxygen removal (Figure 2c). The siloxene bands at 1055 and 2199 cm^−1^, assigned to Si–O–Si and OSi_2_‐Si‐H vibrations, confirm the Kautsky‐type structure [44, 45]. After HCl treatment, the Si–OH‐related band shifts, consistent with protonation of surface hydroxyl groups and subsequent oxygen removal during annealing. Si 2p XPS deconvolution (Figure 2d and Figure S8), acquired after Ar^+^ sputtering to a depth of approximately 100 nm, shows an increased fraction of low‐oxidation‐state silicon (Si^0^, Si^1+^, and Si^2+^) and a decreased fraction of Si^3+^/Si^4+^. Quantitative analysis gives a decrease in oxygen stoichiometry from x = 1.23 for LS‐SiO_x_ to x = 1.01 for DLS‐SiO_x_ (Figure 2e), close to commercial SiO. As the spectra were collected from the subsurface region after penetrating the surface native oxide, they reliably reflect the bulk compositional change and confirm the successful deoxygenation. This installs the thermodynamic variable expected to suppress Li_2_Si_2_O_5_‐rich lithiation products and favor a more Li_4_SiO_4_‐rich, transport‐favorable subsurface during cycling [46, 47].

The modest decrease in surface area and pore size after HCl treatment is particularly important within the stepwise control‐series framework: deoxygenation does not create a highly exposed nanostructure but preserves a compact layered scaffold. This helps avoid the ICE penalty associated with excessive surface exposure while retaining internal voids for strain accommodation. Nitrogen physisorption gives type‐IV adsorption isotherms with H3 hysteresis loops, indicating slit‐like pores between and within the nanolayers (Figure 2f and Figure S9). The specific surface area decreases modestly from 43.58 to 39.03 m^2^ g^−1^ after HCl treatment, and the average pore size decreases from 7.72 to 4.98 nm, consistent with a denser but still porous layered framework. TGA and C 1s XPS further confirm the SWCNT/CMC coating and its loading (Figures S10 and S11), with a calculated SWCNT/CMC loading of 4.5 wt.% and SWCNT loading of 1.8%. Four‐point probe measurements show a pronounced conductivity increase after SWCNT incorporation (Figure S12), verifying that the kinetic/electronic wiring variable is activated.

The DLS‐SiO_x_@CNT thus integrates the three control features, which is hypothesized to establish the conditions for the subsurface to evolve into a Li_4_SiO_4_‐rich, ion‐accessible PSCII during lithiation.

### 2.2 Electrochemical Probing of Deep Alloying and Interfacial Reversibility

Having verified the coupled design at the materials level, we next tested whether its electrochemical signatures follow the predicted order. CV, galvanostatic charge–discharge profiles, *b*‐value analysis, EIS, GITT, and long‐term cycling were used as complementary probes of alloying accessibility, charge‐transfer kinetics, and interfacial reversibility.

**FIGURE 3:**
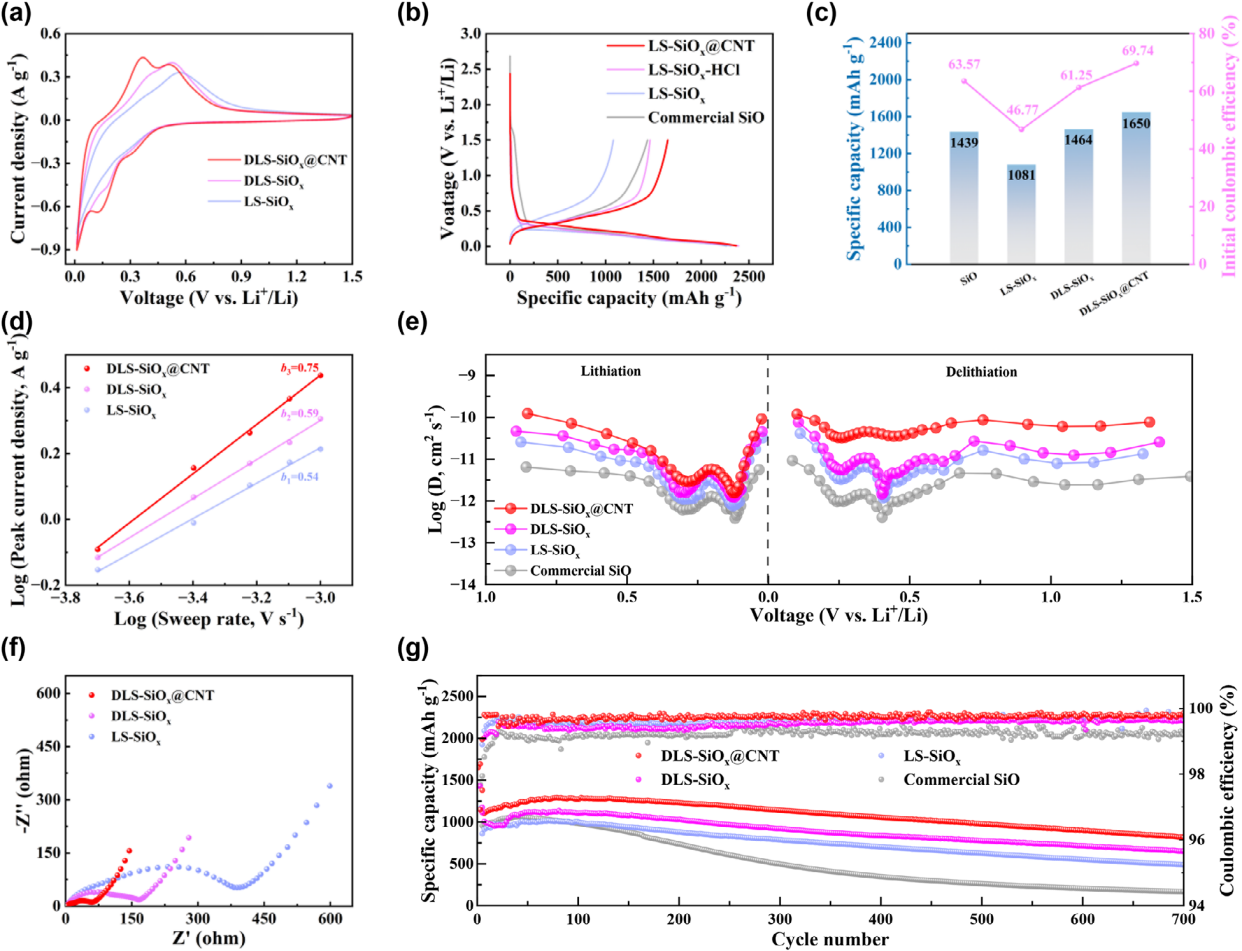
Electrochemical signatures of subsurface phase selection in commercial SiO, LS‐SiO_x_, DLS‐SiO_x_, and DLS‐SiO_x_@CNT. (a) Third‐cycle CV curves of the engineered samples at 0.1 mV s^−1^. (b) Galvanostatic charge–discharge profiles at 0.1 A g^−1^. (c) Initial discharge capacity and ICE, showing the LS‐SiO_x_ ICE penalty and its recovery after deoxygenation and wiring. (d) Linear fitting of log(i)‐log(v) plots; the reported b values represent window‐averaged behavior. (e) GITT‐derived apparent Li^+^ diffusion coefficient versus voltage; the 0.35–0.40 V minimum is assigned to the Li_15_Si_4_‐to‐amorphous‐Si two‐phase dealloying region. (f) EIS after three activation cycles. (g) Long‐term cycling stability and Coulombic efficiency at 1 A g^−1^.

The third‐cycle CVs of the engineered samples show a progressive sharpening of the alloying/dealloying features as the design variables are progressively introduced (Figure 3a; 1st‐cycle CVs and the commercial SiO comparison are shown in Figure S13). LS‐SiO_x_ exhibits broad, low‐intensity redox features and pronounced polarization. DLS‐SiO_x_ shows sharper peaks after oxygen reduction, indicating improved reaction reversibility. DLS‐SiO_x_@CNT displays the most intense and well‐defined peaks, including reduction features near 0.31 and 0.12 V associated with Li‐Si alloying and oxidation peaks near 0.35 and 0.51 V associated with dealloying [30].

Galvanostatic profiles and ICE values reveal why the stepwise comparison is mechanistically informative (Figure 3b,c). Commercial SiO delivers an initial discharge capacity of 1439 mAh g^−1^ with an ICE of 63.57%. Introducing the layered architecture alone lowers both the capacity and ICE for LS‐SiO_x_ (1081 mAh g^−1^ and 46.77%), indicating that the additional surface/interlayer exposure amplifies first‐cycle electrolyte reactions when the composition and electron‐supply control variables are not yet activated. Bulk deoxygenation recovers the capacity to 1464 mAh g^−1^ and the ICE to 61.25% for DLS‐SiO_x_, while SWCNT wiring raises the capacity and ICE further to 1650 mAh g^−1^ and 69.74% for DLS‐SiO_x_@CNT. To rule out capacity contribution from the SWCNT coating itself, a control electrode (SWCNT/CMC) was tested under identical conditions (Figure S14). It delivered only ∼76 mAh g^−1^ with an ICE of ∼51.7%. If the 4.5 wt.% coating had acted merely as a mechanical additive, the composite would deliver a capacity of only ∼1400 mAh g^−1^, substantially lower than the measured 1650 mAh g^−1^. Thus, the layered scaffold alone is not the solution; the full performance emerges only when deoxygenation and interfacial electronic coupling rescue and then exploit that architecture.

The superior initial performance prompted a deeper investigation into the charge‐storage kinetics. To elucidate how the designed features enhance reaction dynamics, a combination of multi‐rate cyclic voltammetry (CV), electrochemical impedance spectroscopy (EIS), and galvanostatic intermittent titration technique (GITT) was employed.

The kinetics of charge storage were quantified by the scan‐rate dependence of the CV peaks (Figure S15 and Figure 3d). The *b*‐values for the delithiation process increase from 0.54 for LS‐SiO_x_ to 0.59 for DLS‐SiOx and 0.75 for DLS‐SiO_x_@CNT, indicating a gradual shift from diffusion‐dominated behavior toward a mixed capacitive/diffusion response. This window‐averaged *b*‐value does not contradict the two‐phase Li_15_Si_4_ feature discussed below; the plateau‐associated region can still show a GITT‐derived minimum even when the overall voltage window exhibits faster mixed kinetics.

GITT provides the most direct electrochemical signature of the alloying pathway opened by the PSCII (Figure 3e and Figure S16, see Supporting Information Experimental Section). DLS‐SiO_x_@CNT exhibits a pronounced apparent‐D_Li+_ minimum near 0.35–0.40 V during delithiation, whereas this feature is much shallower or absent for the samples without full coupling of deoxygenation and electronic interconnection. This minimum corresponds to the Li_15_Si_4_ two‐phase dealloying region, where crystalline Li_15_Si_4_ transforms to amorphous Si and the voltage plateau causes the thermodynamic factor in the GITT‐derived apparent diffusivity to fall sharply. Its depth is consistent with increased accessibility to the Li_15_Si_4_‐associated alloying pathway. The ordering of this feature (DLS‐SiO_x_@CNT > DLS‐SiO_x_ > LS‐SiO_x_ approximately commercial SiO) indicates that deoxygenation plus uniform electron supply opens access to the Li‐rich end of the Si alloying pathway. Bulk deoxygenation reduces Li_2_Si_2_O_5_‐related blocking phases, while the SWCNT network supplies electrons locally so lithiation can proceed beyond only the best‐wired regions of the particle.

EIS provides a direct measure of charge‐transfer resistance (Figure 3f). DLS‐SiO_x_@CNT exhibits the smallest high‐to‐medium‐frequency semicircle, consistent with lower charge‐transfer resistance after SWCNT wiring. Analysis of the low‐frequency Warburg region revealed that the apparent Li^+^ diffussion coefficient *D*_app, Li+_ for DLS‐SiO_x_@CNT, is approximately 7.2 times higher than that of LS‐SiO_x_ (Figure S17), showing that the full design reduces both electronic and ionic transport limitations.

Long‐term cycling further follows this series (Figure 3g and Figure S18). After three activation cycles at 0.1 A g^−1^, cells were cycled at 1 A g^−1^. All materials exhibited an initial capacity increase during the first 100 cycles, which is commonly observed in silicon‐based anodes and can be attributed to the gradual electrode activation process [48, 49]. Commercial SiO decays rapidly, retaining less than 20% of its capacity after 700 cycles due to particle pulverization and massive volume swings [9, 50]. LS‐SiO_x_ retains 487 mAh g^−1^, reflecting the benefit of the layered framework but also its limited phase control. DLS‐SiO_x_ reaches a maximum capacity of 1116 mAh g^−1^ and retains 651 mAh g^−1^ after 700 cycles, consistent with the added benefit of deoxygenation. DLS‐SiO_x_@CNT retains over 820 mAh g^−1^ after 700 cycles, showing that the full PSCII design sustains both high capacity and interfacial stability.

The CE data provide the corresponding interfacial‐reversibility signature (Figure S19). Commercial SiO requires more than 15 cycles to exceed 99% CE and stabilizes near 99.1%. LS‐SiO_x_ reaches approximately 99.5% within ten cycles, whereas DLS‐SiO_x_ stabilizes near 99.3%, likely reflecting residual side reactions during activation of the deoxygenated layered structure. DLS‐SiO_x_@CNT reaches 99.8% after only eight cycles and maintains an average CE above 99.6% during extended cycling. The convergence of capacity retention, CE, EIS, and GITT establishes that performance improvement is not a standalone metric but a consequence of redirecting subsurface phase selection and electron supply together.

### 2.3 Post‐Cycling Evidence for PSCII Formation and Subsurface Phase Selection

**FIGURE 4:**
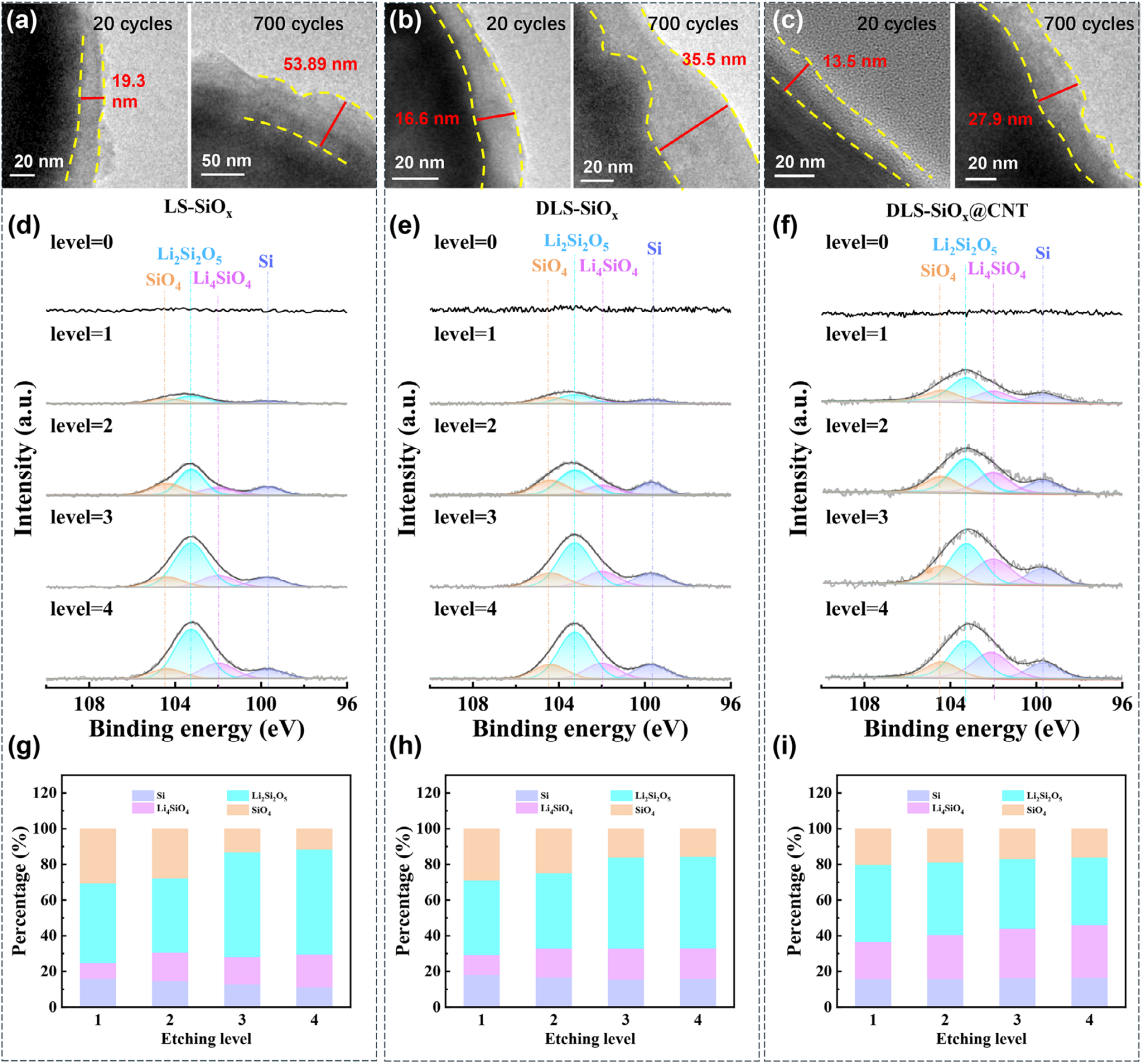
Direct chemistry of the post‐cycling subsurface. (a–c) Cross‐sectional TEM images after 20 and 700 cycles for (a) LS‐SiO_x_, (b) DLS‐SiO_x_, and (c) DLS‐SiO_x_@CNT. (d–f) Si 2p XPS depth profiles for (d) LS‐SiO_x_, (e) DLS‐SiO_x_, and (f) DLS‐SiO_x_@CNT; depth increases downward from the cycled surface toward the bulk. Quantitative phase fractions of different types of Si in (g) LS‐SiO_x_, (h) DLS‐SiO_x_, and (i) DLS‐SiO_x_@CNT.

Post‐cycling analysis was then performed to test whether the stepwise electrochemical trends correlate with chemical and morphological changes in the cycled subsurface. SEM shows that DLS‐SiO_x_@CNT remains mechanically coherent after 700 cycles, in contrast to the fractured commercial SiO electrode (Figure S20). This mechanical stability is further supported by the electrode thickness expansion (0.5% for DLS‐SiO_x_@CNT versus 20% for commercial SiO, with 1.4% for LS‐SiO_x_ and 0.72% for DLS‐SiO_x_, Figure S21) and by AFM QNM measurements, which reveal a significantly higher and more uniform Young's modulus for DLS‐SiO_x_@CNT, confirming the formation of a mechanically robust SEI (Figure S22). Cross‐sectional TEM directly measures SEI evolution (Figure 4a–c). The SEI on DLS‐SiO_x_@CNT grows from 13.5 nm after 20 cycles to only 27.9 nm after 700 cycles, thinner than that on LS‐SiO_x_ (19.3–53.89 nm) and DLS‐SiO_x_ (16.6–35.5 nm), and far thinner than that on commercial SiO (43.16–194.8 nm, Figure S23). This SEI‐thickness trend provides the morphological counterpart to the CE evolution.

XPS depth profiling directly probes the cycled subsurface chemistry that defines the PSCII (Figure 4d–f and Figure S21). LS‐SiO_x_ shows a Li_2_Si_2_O_5_‐rich subsurface whose blocking character is consistent with its thicker SEI and weaker GITT Li_15_Si_4_ signature [51]. Quantitative phase fractions derived from the XPS deconvolution confirm that LS‐SiO_x_ exhibits a Li_2_Si_2_O_5_‐dominated subsurface across all etching depths, whereas DLS‐SiO_x_ shows reduced Li_2_Si_2_O_5_ and increased Li_4_SiO_4_(Figure 4g‐i). Notably, DLS‐SiO_x_@CNT exhibits the highest Li_4_SiO_4_ content and the lowest Li_2_Si_2_O_5_ content across the entire probed depth range, quantitatively confirming that the integrated PSCII design pushes the distribution further toward a Li_4_SiO_4_‐rich, Li_2_Si_2_O_5_‐suppressed subsurface. This trend is consistent with the hypothesized phase‐selection mechanism. This trend supports the proposed phase‐selection hypothesis, namely oxygen stoichiometry sets the thermodynamic tendency for silicate formation, while the SWCNT network homogenizes local electron accessibility during lithiation, so the phase selection occurs more uniformly across the particle. FIB‐EDX cross sections provide an independent compositional check (Table S1), with Si/O atomic ratios following DLS‐SiO_x_@CNT (0.56) > LS‐SiO_x_ (0.38) > commercial SiO (0.12), confirming that the engineered electrode better preserves a Si‐rich, deoxygenated internal composition.

**FIGURE 5:**
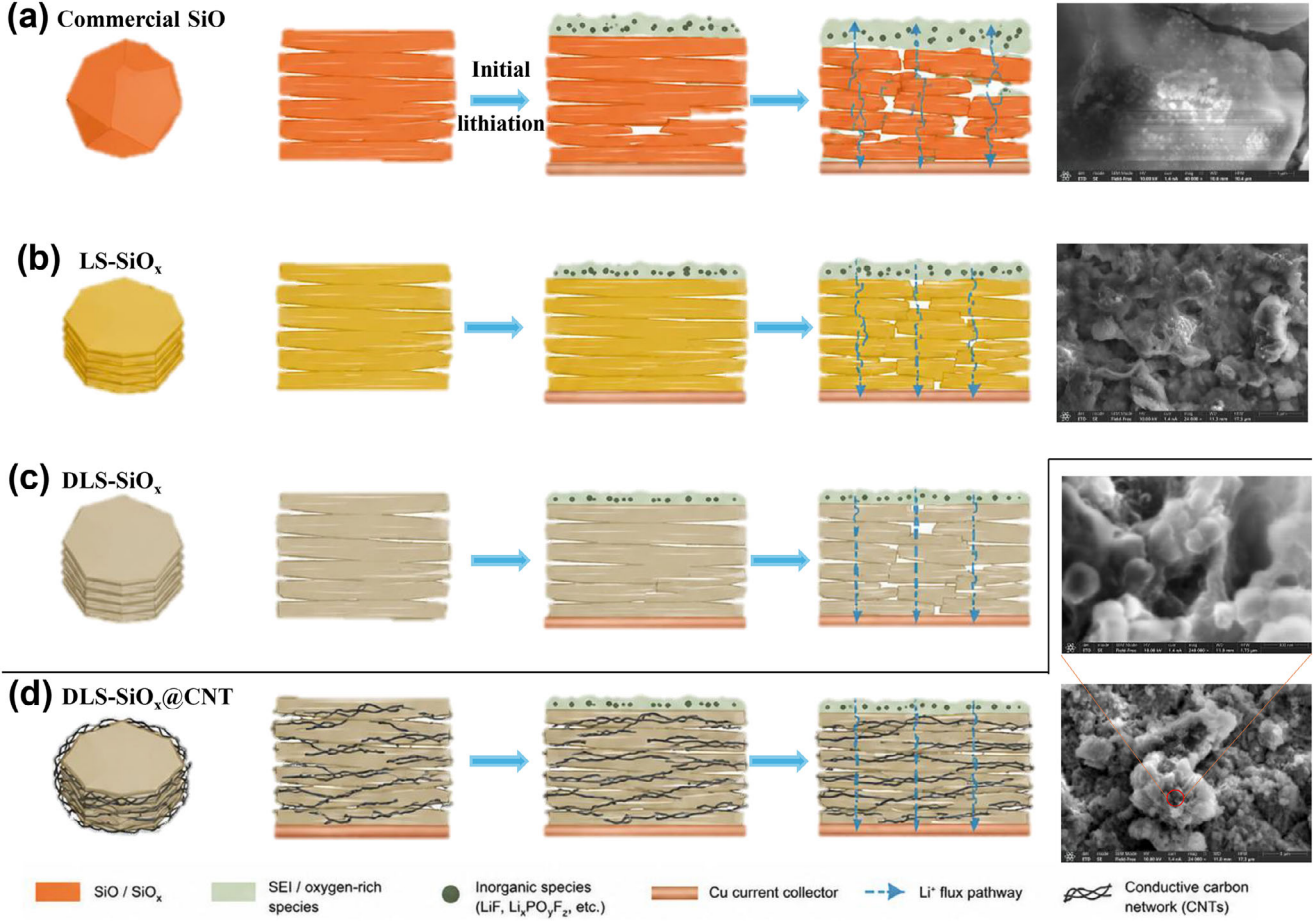
Schematic illustration of structural and chemical evolution during cycling. Rows compare commercial (a) SiO, (b) LS‐SiO_x_, (c) DLS‐SiO_x_, and (d) DLS‐SiO_x_@CNT; columns compare the pristine state, first lithiation, and extended cycling after 700 cycles. The right column shows SEM images after ion milling, highlighting the increasing preservation of internal morphology along the coupled design.

The mechanistic schematic in Figure 5 integrates these observations across the coupled design. Commercial SiO undergoes severe cracking and uncontrolled SEI thickening. LS‐SiO_x_ benefits from layered buffering but retains a Li_2_Si_2_O_5_‐rich subsurface that limits transport. DLS‐SiO_x_ suppresses part of this blocking chemistry through deoxygenation. DLS‐SiO_x_@CNT combines deoxygenation with conductive wiring, forming a Li_4_SiO_4_‐rich, electronically connected subsurface and the thinnest SEI. Taken together, the SEI‐thickness trend, the subsurface phase‐fraction trend, the GITT 0.35–0.40 V signature, and the preserved Si/O ratio all rank the samples in the same order, providing independent confirmations of the PSCII picture.

The precision etching and coating system (PECS) ion‐milled SEM images corresponding to the schematic figure in Figure 5 provide an inner structural counterpart to the chemical analysis. SiO shows disordered particles after cycling, while LS‐SiO_x_ shows heterogeneous reconstruction with aggregated domains, collapsed pores, and locally densified regions, consistent with nonuniform stress propagation and Li_2_Si_2_O_5_‐rich accumulation. DLS‐SiO_x_@CNT retains a more homogeneous porous‐conductive framework without large‐scale collapse, indicating that conductive network formation and deoxygenation stabilize not only the outer SEI but also the cycled subsurface interior.

This convergence across morphology, chemistry, and electrochemistry is the central evidence for the PSCII: the engineered sample does not merely add a conductive coating to SiO_x_, but redirects the first‐lithiation subsurface chemistry into a kinetically accessible region that remains structurally connected during long cycling.

### 2.4 Extended Performance and Practical Validation of the PSCII Engineered Anode

After establishing the phase‐selection mechanism, we evaluated whether it translates into rate capability, long cycling, and full‐cell operation.

**FIGURE 6:**
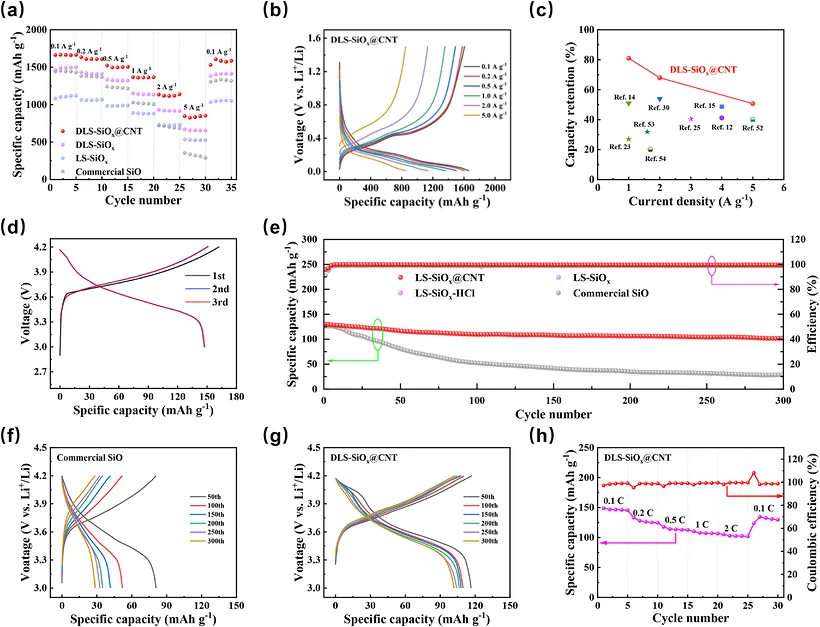
Performance outcomes and practical validation. (a) Rate performance of LS‐SiO_x_, DLS‐SiO_x_, DLS‐SiO_x_@CNT, and commercial SiO. (b) Charge–discharge curves of DLS‐SiO_x_@CNT at different current densities. (c) Literature comparison of rate capability for Si‐based anodes, highlighting DLS‐SiO_x_@CNT. (d) Charge–discharge curves of the DLS‐SiO_x_@CNT//LCO full cell. (e) Full‐cell cycling performance and CE of DLS‐SiO_x_@CNT//LCO and commercial SiO//LCO cells. (f,g) Charge–discharge curves of DLS‐SiO_x_@CNT//LCO and SiO//LCO full cells during cycling at 0.2 C. (h) Rate performance of the DLS‐SiO_x_@CNT//LCO full cell.

Rate performance follows directly from the transport picture (Figure 6a,b and Figure S26). Commercial SiO retains only about 20% of its low‐rate capacity at 5 A g^−1^, with nearly vanished charge/discharge plateaus because ion/electron transport is insufficient at high current. LS‐SiO_x_ improves rate response through shorter diffusion lengths in the layered architecture. DLS‐SiO_x_ performs better across the rate series after deoxygenation. DLS‐SiO_x_@CNT gives the highest rate capability, delivering approximately 830 mAh g^−1^ at 5 A g^−1^, corresponding to about 50% of its low‐rate capacity, and recovering when the current returns to 0.1 A g^−1^. The literature comparison highlights that this high‐rate response is achieved together with high reversible capacity (Figure 6c) [12, 14, 15, 23, 25, 30, 52, 53, 54]. Long‐term cycling at 2 A g^−1^ further shows over 85% capacity retention after 1000 cycles (Figure S23), supporting the durability of the same mechanism under more demanding current density.

Full cells were assembled using a prelithiated DLS‐SiO_x_@CNT anode and commercial LiCoO_2_ (LCO) cathode within 3.0–4.2 V, with capacities reported based on cathode mass and electrode balancing described in the Experimental Section. The DLS‐SiO_x_@CNT//LCO cell delivers 141 mAh g^−1^ at 0.1 C and retains 103 mAh g^−1^ at 2 C (Figure 6d,h). During cycling at 0.2 C, it retains 102 mAh g^−1^ after 300 cycles, whereas the commercial SiO//LCO control decays to 28 mAh g^−1^ under the same conditions (Figure 6e–g). These full‐cell data should be interpreted as a device‐level consequence of the stabilized subsurface chemistry: less continuous lithium consumption, more stable voltage profiles, and better preservation of accessible Si capacity.

## 3 Conclusion

This paper identifies subsurface lithium silicate phase selection as a critical and hidden control factor governing the performance of high‐capacity SiO_x_ anodes. It is proposed that bulk deoxygenation and uniform electron supply can bias the first‐lithiation product distribution away from Li_2_Si_2_O_5_‐rich blocking phases and toward a Li_4_SiO_4_‐rich, ion‐accessible subsurface—a hypothesis termed the Phase‐Selective Conductive Interfacial Interlayer (PSCII). To test this hypothesis, a stepwise material series was designed in which layered buffering, oxygen stoichiometry, and conductive wiring were progressively activated. Through a systematic and stepwise design approach, we demonstrate that layered architectures enhance mechanical buffering but sacrifice initial Coulombic efficiency. Bulk deoxygenation effectively shifts lithium silicate chemistry away from Li_2_Si_2_O_5_‐rich blocking phase, while SWCNT conductive wiring enables more uniform local lithiation. The fully engineered DLS‐SiO_x_@CNT electrode develops a Li_4_SiO_4_‐rich, transport‐permissive PSCII, as confirmed by XPS depth profiling, the Li_15_Si_4_‐associated GITT signature near 0.35–0.40 V, minimal SEI growth, and preservation of Si‐rich composition after cycling. This mechanism produces a balanced performance profile: 1650 mAh g^−1^ reversible capacity, ∼830 mAh g^−1^ at 5 A g^−1^, >820 mAh g^−1^ after 700 cycles at 1 A g^−1^, 85% capacity retention after 1000 cycles at 2 A g^−1^, and stable LCO full‐cell operation. The proposed phase‐selective mechanism remains a hypothesis derived from experimental observations and literature on lithium silicate ionic transport properties, and the mechanistic picture presented here is intended to inspire future computational studies to further validate or refine the underlying thermodynamic arguments. The broader message is that durable SiO_x_ anodes require coordinated control of bulk oxygen stoichiometry, subsurface phase chemistry, and local electron supply, rather than conductivity enhancement or morphology design alone.

## Author Contributions

W.P. and Z.C. proposed the research. Z.C. performed the experiments and characterized the materials. W.D., H.Q., and X.Z. analyzed the electrochemical data. W.P. performed the Cryo‐FIB‐SEM tests. Y.W. and C.W. performed the mechanistic analysis. W.P. and D.Y.C.L. supervised the project. W.P. and Z.C. wrote the paper. Y.Z., Y.J., D.Y.C.L., M.D., and H.W. revised the paper. All authors engaged in the discussion of the results.

## Conflicts of Interest

The authors declare no conflicts of interest.

## Supporting information




**Supporting File**: advs77220‐sup‐0001‐SuppMat.docx.

## Data Availability

The data that support the findings of this study are available from the corresponding author upon reasonable request.
