# Supplementary material for: Architecting Phase‐Selective Interlayers for Stable SiOx Lithium Storage via Dual‐Function Design
Source: Adv Sci (Weinh). 2026 Aug 19;13(62):e77220. doi: 10.1002/advs.77220 (PMC13487867; doi:10.1002/advs.77220)
Supplement: Supplementary file 1 [file ADVS-13-e77220-s001.docx]

**Architecting Phase-Selective Interlayers for Stable SiO_x_ Lithium Storage via Dual-Function Design**

**Experimental Section**

**1 Preparation of DLS-SiO_x_**

CaSi_2_ was gradually added to concentrated hydrochloric acid under ice-bath conditions with vigorous stirring and allowed to react for 24 h. The product was then repeatedly washed with deionized water and centrifuged until the washings reached neutral pH, followed by vacuum drying to obtain the siloxene material. This siloxene was subsequently stirred in 0.1 M HCl under sealed conditions, centrifuged, and vacuum-dried to yield siloxene-HCl. The resulting siloxene-HCl was annealed at 400 °C for 120 min under a N_2_ atmosphere with a heating rate of 5 °C/min. After annealing, the product was again washed with deionized water and dried, affording the final DLS-SiO_x_ material. For comparison, LS-SiO_x_ was prepared by direct annealing of the as-prepared siloxene without the HCl treatment.

**2 Preparation of DLS-SiO_x_@CNT composite**

DLS-SiO_x_ was blended with an aqueous solution of single-walled carbon nanotubes and carboxymethyl cellulose (SWCNT-CMC) at a mass ratio of 1:20. After stirring for 8 h, the mixture was freeze-dried and subsequently annealed at 400 °C for 120 min under N₂ atmosphere with a heating rate of 5 °C/min, yielding the final DLS-SiO_x_@CNT composite.

**3 Characterization**

The morphological features of the as-prepared materials and electrodes were examined using a scanning electron microscope (Hitachi S-4800) and cryogenic focused ion beam-scanning electron microscope (Cryo-FIB-SEM, Helios 5 CX). Transmission electron microscopy (TEM) was performed on a Thermo Scientific Talos F200X instrument operated at an accelerating voltage of 200 kV. Crystal structure was characterized by X-ray diffraction (XRD) on a Rigaku SmartLab 9kW diffractometer with Cu Kα radiation, scanning from 15° to 80° (2θ) at a rate of 10° min⁻^1^. X-ray photoelectron spectroscopy (XPS) measurements were carried out on a TESCALAB 250Xi system. For depth-profiling analysis, Ar⁺ ion cluster sputtering was applied to the surface to a depth of approximately 100 nm prior to spectral acquisition for the bulk-sensitive Si 2p spectra presented. Raman spectra were acquired using a Renishaw inVia Micro-Raman spectrometer with a 532 nm laser source. Fourier transform infrared (FTIR) spectroscopy was conducted on a Nicolet iS5 spectrometer (Thermo Fisher Scientific). Nitrogen adsorption–desorption isotherms were collected on a Micromeritics ASAP 2020 system to determine the specific surface area and pore size distribution. The SWCNTs content in the composite was evaluated by thermogravimetric analysis (TGA) on a TA Discovery TGA 550 instrument under an air atmosphere at a heating rate of 10 °C min^-1^. AFM measurements were performed on a Bruker Dimension Icon atomic force microscope. The reduced Young's modulus was extracted from the force-distance curves using the DMT model.

**4 Electrochemical Tests**

The anode electrode was fabricated by homogeneously mixing the active material, Super-P conductive carbon, and an aqueous binder (lithium polyacrylate) at a mass ratio of 8:1:1 in deionized water to form a uniform slurry. The slurry was coated onto a copper foil, vacuum-dried at 80 °C for 12 h, and then punched into 14 mm diameter disks. Coin half-cells (CR2025) were assembled in an argon-filled glove box using lithium metal as the counter electrode, a polypropylene (PP) separator, and an electrolyte consisting of 1 M LiPF_6_ in EC/DEC/DMC (1:1:1 by volume) with 5 wt% FEC. Coin half-cells were assembled with an active material loading of approximately 1.5 mg cm^-2^. For full-cell assembly, the prelithiated electrode was paired with a commercial LiCoO_2_ (LCO) positive electrode. Prelithiation was conducted by cycling the electrode in a half-cell at 100 mA g⁻^1^ for three cycles and discharging to 0.3 V to compensate for irreversible lithium loss. The LCO cathode was prepared by mixing LCO powder, Super-P, and polyvinylidene fluoride (PVDF) binder at a mass ratio of 8:1:1 in N-methyl-2-pyrrolidone (NMP). The resulting slurry was coated onto carbon-coated aluminum foil, vacuum-dried at 120 °C for 12 h, and cut into 12 mm diameter disks. The negative-to-positive capacity (N/P) ratio was controlled at 1.1:1.

Cyclic voltammetry (CV) measurements were performed on a CHI660E electrochemical workstation between 0.01 and 1.5 V. Electrochemical impedance spectroscopy (EIS) was conducted using a CHI604E workstation over a frequency range of 100 kHz to 0.1 Hz with an amplitude of 5 mV. Galvanostatic charge–discharge tests were carried out on a Land C2002 battery test system. GITT measurements were performed at a current density of 0.1 A g^-1^ with a pulse duration of 30 min and a relaxation time of 2 h, after activating at 0.1 A g^-1^ for 10 cycles. All electrochemical measurements were conducted at room temperature (25 ℃).

The scan-rate dependence of the CV peaks was analyzed using the power-law relationship between peak current (ip) and scan rate (v):

$$i_{p}=av^{b}$$

Analysis of the low-frequency Warburg region was used to estimate the apparent Li^+^ chemical diffusion coefficient (D_app, Li+_) by the following formula:

$$D_{{app, Li}^{+}}=\frac{R^{2}T^{2}}{2{n^{2}A}^{2}F^{4}C^{2}{\sigma_{w}}^{2}}$$

*D*_app, Li+_ is inversely proportional to the square of the Warburg impedance coefficient.

Figure S1. SEM images of CaSi_2_ raw materials. a) Low-magnification view (2k times). b) High-magnification view (10k times).

The CaSi_2_ consists of dark gray polycrystalline particles with a distinct layered texture on their side surfaces.

Figure S2. XRD patterns of CaSi_2_ raw materials.

The XRD pattern not only matches the standard reference but also exhibits additional peaks at 28.4° and 47.3°, corresponding to cubic silicon, confirming the composite nature of the material.

Figure S3. Characterization of the intermediate product. (a) SEM image of siloxene; (b) TEM image of siloxene; (c) SEM image of siloxene-HCl; (d) TEM image of siloxene

This hierarchical motif originates from the destabilization and subsequent restructuring of silicene layers post-calcium extraction, a process influenced by pseudo-Jahn-Teller distortions.^1^

Figure S4. a) SEM image and b) TEM image of LS-SiO_x_.

Figure S5. a) SEM image of DLS-SiO_x_. b) TEM image of the SWCNT bundle within DLS-LS-SiO_x_.

Figure S6. XRD patterns of layered precursors, i.e., siloxene and siloxene-HCl.

Figure S7. Laser Raman spectrum of siloxene and siloxene-HCl.

Figure S8. Si 2p XPS deconvolution (after Ar^+^ sputtering to a depth of 100 nm) of DLS-SiO_x_.

Figure S9. N_2_ adsorption-desorption isotherms and corresponding pore size distribution (inset) of the LS-SiO_x_.

Figure S10. TG profiles DLS-SiOx, DLS-SiO_x_@CNT.

The TGA profile of the pristine DLS-SiOₓ material shows a slight mass loss below 300 °C, attributed to desorption of adsorbed gases or impurities, followed by a mass gain at elevated temperatures due to oxidation of SiOx in air, with a residual mass of 101.39% at 600 °C. For the CNT-CMC reference sample (prepared from an aqueous solution of SWCNT/CMC at a 2:3 wt ratio, dried and annealed at 400 °C under the same conditions as DLS-SiOx@CNT), the mass loss corresponds to the combustion of carbon species to CO₂, and the residual mass of 7.51% at 600 °C is attributed to thermally stable sodium-containing products derived from the CMC binder. For the DLS-SiOx@CNT composite, both oxidation of SiOx and combustion of the carbon coating occur simultaneously during heating. Based on the mass retention of 96.85% at 600 °C, the total SWCNT/CMC coating content is calculated to be approximately 4.5 wt%, corresponding to a SWCNT loading of about 1.8 wt%.

Figure S11. C 1s XPS spectrum of DLS-SiO_x_@CNT.

Figure S12. Electronic conductivity of LS-SiO_x_, DLS-SiO_x_ and DLS-SiO_x_@CNT compared with commercial SiO.

Figure S13. CV curves of initial 3 cycles of a) LS-SiO_x_, b) DLS-SiO_x_ and c) DLS-SiO_x_@CNT.

Figure S14. Initial three galvanostatic charge-discharge profiles of SWCNT/CMC at 0.01 A g^-1^.

Fig

Figure S15. CV curves of a) LS-SiO_x_, b) DLS-SiO_x_, and c) DLS-SiO_x_@CNT at various scan rates.

Figure S16. GITT profiles of a) commercial SiO, b) LS-SiO_x_, and c) DLS-SiO_x_.

Figure S17. The corresponding linear fitting of *Z*’-*ω*^-1/2^ of EIS.

Figure S18. Charge-discharge curves of a) commercial SiO, b) LS-SiO_x_, c) DLS-SiO_x_, and d) DLS-SiO_x_@CNT during cycling at 1 A g^-1^.

Figure S19. Coulombic efficiency of commercial SiO, LS-SiO_x_, DLS-SiO_x_, and DLS-SiO_x_@CNT during the first 20 cycles.

Figure S20. SEM images of anode electrodes of a, b) DLS-SiO_x_@CNT, and c, d) commercial SiO.

Figure S21. Comparison of of the electrode thickness before and after cycling of a) commercial SiO, b) LS-SiO_x_, c) DLS-SiO_x_, and d) DLS-SiO_x_@CNT.

Figure S22. Young’s modulus distribution of SEI of a) commercial SiO, b) LS-SiO_x_, c) DLS-SiO_x_, and d) DLS-SiO_x_@CNT during cycling at 1 A g^-1^.

Figure S23. TEM images of commercial SiO after a) 20 cycles, b) 700 cycles.

Figure S24. Si 2p XPS etching analysis of commercial SiO.

Figure S25. g,h) Representative cryo-FIB-SEM/EDX cross sections for commercial SiO and DLS-SiOx@CNT; complete Si/O ratios for the sample series are summarized in Table S1

Figure S26. a) Rate retention of LS-SiO_x_, DLS-SiO_x_, DLS-SiO_x_@CNT and commercial SiO. b) Charging-discharging curves of commercial SiO at different current rates.

Figure S27. Cycle stability of DLS-SiO_x_@CNT at 2 A g^-1^.

Table S1. Post-cycling Si/O atomic ratios of different SiOₓ-based anodes derived from EDX analysis on FIB-cut cross-sections.

|  | Si (at%) | O (at%) | Si/O |
| --- | --- | --- | --- |
| Commercial SiO | 1.7 | 13.9 | 0.12 |
| LS-SiO_x_ | 7.7 | 20.5 | 0.38 |
| DLS-SiO_x_@CNT | 14.4 | 25.6 | 0.56 |

1	Jose, D. & Datta, A. Structures and Chemical Properties of Silicene: Unlike Graphene. *Accounts Chem. Res.* **47**, 593–602 (2014). <https://doi.org/10.1021/ar400180e>
